# Therapeutic Hypothermia Modifies Perinatal Asphyxia-Induced Changes of the Corpus Callosum and Outcome in Neonates

**DOI:** 10.1371/journal.pone.0123230

**Published:** 2015-04-29

**Authors:** Thomas Alderliesten, Linda S. de Vries, Yara Khalil, Ingrid C. van Haastert, Manon J. N. L. Benders, Corine Koopman-Esseboom, Floris Groenendaal

**Affiliations:** Department of Neonatology, Wilhelmina Children′s Hospital, University Medical Center Utrecht, Utrecht, The Netherlands; Robert Debre Hospital, FRANCE

## Abstract

**What Is Known about this Subject?:**

Diffusion-weighted MRI has demonstrated changes in the corpus callosum of term neonates with perinatal asphyxia. The severity of cerebral changes demonstrated using diffusion-weighted MRI is difficult to assess without measuring values of the Apparent Diffusion Coefficient (ADC).

**What Is New?:**

ADC values of the anterior part of the corpus callosum are slightly higher than of the posterior part in full term infants with perinatal asphyxia. Low ADC values of the corpus callosum were associated with an adverse outcome in infants with perinatal asphyxia. In infants treated with hypothermia lower ADC values than with normothermia were associated with a poor outcome, supporting neuroprotective effects of hypothermia

**Background:**

Using MRI, changes can be detected in the corpus callosum (CC) following perinatal asphyxia which are associated with later neurodevelopmental outcome.

**Aim:**

To study the association between the apparent diffusion coefficient of water (ADC) in the CC on MRI in neonates with perinatal asphyxia and neurodevelopmental outcome at 18 months of age.

**Subjects, Methods:**

Of 121 infants 32 (26%) died and 13 (11%) survived with an adverse neurological outcome. Sixty-five (54%) received therapeutic hypothermia. MRI was performed within 7 days after birth using a 1.5 T or 3.0 T system, and ADC values were measured in the anterior and posterior CC. The association between ADC and composite outcome (death or abnormal neurodevelopment) was analyzed for both normothermia and hypothermia cases using receiver operating characteristics.

**Results:**

ADC values of the posterior CC were lower than of the anterior part (mean difference 0.050 x 10^-3^ mm^2^/s, p<0.001). Field strength did not affect ADC values. ADC values of the posterior part of the CC were significantly lower in infants with basal ganglia/thalamus or near total brain injury (p<0.001). Lower ADC values were associated with an adverse outcome, but cut-off levels were lower after hypothermia (1.024 x 10^-3^ mm^2^/s vs 0.969 x 10^-3^ mm^2^/s)

**Conclusion:**

Low ADC values of the posterior part of the corpus callosum are associated with an adverse outcome in term or near term neonates with perinatal asphyxia. Therapeutic hypothermia slightly modifies this association, showing that lower values were needed for an adverse outcome.

## Introduction

Perinatal asphyxia is still common in the more affluent parts of the world and is known to have adverse effects on the brain of the full term neonate. Hypoxic-ischemic injury can be visualized using MRI.[1] In spite of the use of therapeutic hypothermia for neuroprotection still 45–50% of the full term neonates with perinatal asphyxia either die or have an adverse neurodevelopmental outcome caused by irreversible brain injury.[2]

In a previous study we have demonstrated that the surface area of the corpus callosum (CC) is reduced in 9–10 year old children who suffered perinatal asphyxia followed by encephalopathy.[3] This reduced callosal surface, especially of the splenium, was associated with a significantly higher Total Impairment Score on the Movement Assessment Battery for Children. Others have reported that the size of the CC of adolescents was reduced, and the structure was altered after perinatal asphyxia.[4, 5] Furthermore, CC size correlated with cognitive function. [4]

Recently, two publications have reported restricted diffusion in the CC in term infants who were resuscitated after perinatal asphyxia.[6, 7] However, the severity of this restricted diffusion may be difficult to detect, especially in infants with substantial brain injury who show multiple areas of restricted diffusion throughout the brain.[1, 8] Therefore, we have suggested in a recent paper that Apparent Diffusion Coefficient (ADC) measurements should be performed in regions of interest (ROI) for quantification of Diffusion Weighted (DW)-MRI.[9]

We hypothesized that low ADC values of the anterior and posterior part of the CC in term infants with perinatal asphyxia were associated with an adverse neurological outcome.

Since these ADC changes pseudo-normalize from the end of the first week onward after the hypoxic-ischemic event, we focused in the present study on infants who had an MRI within 7 days after the presumed hypoxic-ischemic event (i.e. birth).[9–11]

As the association between callosal changes and outcome might be modified by therapeutic hypothermia, we compared infants with and without hypothermia.

## Subjects

In the period 1 January 2003 to 31 December 2012 a total of 233 infants with perinatal asphyxia were admitted to our level III NICU. Perinatal asphyxia was defined as described previously.[12] Infants with congenital malformations or syndromes, infants who died before an MRI could be performed, and infants who had an MRI after day 7 of life were excluded. In total 121 (52%) patients were eligible for the study. Details are presented in Table 1.

All infants had routine sequential cranial ultrasound examinations, and aEEG monitoring during the first 96 hours after admission.

Since January 2008 whole body hypothermia was used for neuroprotection in term infants with perinatal asphyxia and neonatal encephalopathy. From the 121 studied patients, 65 (53.7%) were treated with hypothermia.

**Table 1:** Clinical data of the study patients.

	Normothermia	Hypothermia	P-value
	n = 56	n = 65	
Gestational age (mean ± s.d.)	40.1 ± 1.6	40.2 ± 1.4	0.70
Birth weight (mean ± s.d.)	3239 ± 469	3553 ± 565	0.001
pH (mean ± s.d.)	6.99 ± 0.18	6.95 ± 0.21	0.35
Apgar 1 min (median, IQR)	2 (3)	2 (2)	0.08
Apgar 5 min (median, IQR)	5 (2)	4 (3)	0.006
Sarnat (n, %)			0.03
Grade I	2 (3.5)	6 (9.2)	
Grade II	51 (91.1)	47 (72.3)	
Grade III	3 (5.3)	12 (18.5)	
Age at MRI (hours; mean ± s.d.)	98 ± 35	112 ± 30	0.023
MRI pattern (n, %)			0.028
Normal	13 (23.2)	28 (43.1)	
Watershed	15 (26.8)	16 (24.6)	
Basal ganglia/ thalamus	10 (17.9)	13 (20.0)	
Near total	18 (32.1)	8 (12.3)	
Outcome (n, %)			0.007
Favorable	29 (51.8)	47 (72.3)	
Survived, adverse	11 (19.6)	2 (3.1)	
Died	16 (28.6)	16 (24.6)	

## Methods

### MRI

MRI was performed preferably within day 4–7 after the hypoxic-ischemic event using a 1.5 or 3.0 T Philips system (Philips Medical Systems, Best, The Netherlands). MRI included sagittal T1 weighted, and axial T2-weighted images, as well as axial T1 (3.0 T) or Inversion Recovery (1.5 T) weighted axial images. All axial images have 2 mm slice thickness without a gap. In addition, an echo-planar imaging technique was used for DW imaging, 4-mm-thick sections, 0-mm section gap, and *b*-values of 0 and 1000 sec/mm^2^ (1.5 T) or 800 sec/mm^2^ (3.0 T). These *b*-values were used in standard DWI scanning protocols.

The severity of brain injury as seen on MRI was graded as normal, predominantly watershed lesions, predominantly lesions to the basal ganglia or thalamus, or ‘near-total’ brain injury indicating severe injury to both white and grey matter, as described previously.[13, 14] Trace ADC maps were generated on the basis of DW images acquired over the three orthogonal axes (trace ADC = [X + Y + Z]/3).

**Fig 1:**
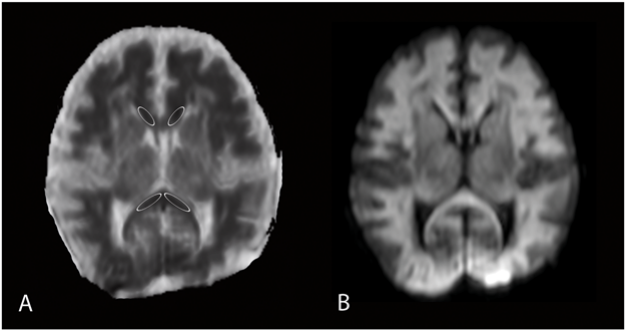
Placement of ROIs in the corpus callosum of a neonate with ‘near- total’ brain injury. ADC values of the left and right side of the corpus callosum were averaged. A) ADC-map and B) corresponding diffusion weighted image.

Image post processing was performed by drawing ROIs in the left and right anterior and posterior parts of the CC on an axial slice of the ADC map (see Fig 1). ADC values were extracted, and left and right values were averaged.

### Assessment of neurodevelopmental outcome

Surviving infants were seen in our follow-up clinic at 3, 9, and 18 months of age. Follow-up data obtained at 18 months were used for analysis of neurodevelopmental outcome.

Neurodevelopment was assessed using the Griffiths mental development scales (GMDS).[15] A Griffiths DQ below 85 was considered adverse. Cerebral palsy (CP) was diagnosed according to the definition and classification described by Rosenbaum et al.[16, 17] and the level of gross motor function was scored according to the gross motor function classification system (GMFCS).[17] Both low DQ scores and/or CP were considered survival with an adverse neurodevelopment. Death or survival with an adverse neurodevelopment were taken as an adverse composite outcome.

### Statistical analysis

All measurements were performed by the same person (YK). Intra-observer consistency was assessed by measuring 10 infants twice. ADC values differed less than 2%.

ADC values of anterior and posterior parts, were compared using paired t-tests. A p-value of <0.05 was considered statistically significant. Results of different field strengths and *b*-values were assessed by comparing ADC values of the patients with a good outcome using t-tests. ADC values of the posterior CC were analyzed using linear modelling, with the interaction of MRI pattern of injury and hypothermia and their interaction as dependent variables.

Finally, receiver operating characteristic (ROC) analysis of ADC values of the posterior CC was performed in normothermia and hypothermia cases to find cut-off levels of ADC values.

Power analysis demonstrated that a difference in the ADC values of 20% between the groups with a good and an adverse outcome could be detected in our infants with a power of more than 0.90.

This study was reviewed and approved by our institutional ethics committee. For this type of study, no informed consent is required in our country.

## Results

Clinical data of the infants are presented in Table 1. Mortality was 28.6% in the normothermic group and 24.6% in the hypothermic group, whereas an adverse composite outcome was seen in 48.2%, and 27.7%, respectively. Differences in adverse outcome between the era of hypothermia and before the introduction of hypothermia were significant (p = 0.02).

The dataset is available as supporting information (S1 Dataset).

### Field strength and ADC values

Results are presented in Table 2. Differences between ADC values obtained at 1.5 T (*b*-value 1000 sec/mm^2^) and 3.0 T (*b*-value 800 sec/mm^2^) were not significant.

**Table 2:** Field strength and ADC values (x 10^-3^ mm^2^/s) of the anterior and posterior parts of the corpus callosum in infants with a normal outcome.

	1.5 T, *b*-value 1000 sec/mm^2^	3.0 T, *b*-value 800 sec/mm^2^	P-value
Anterior CC	1.096 ± 0.145	1.085 ± 0.075	0.65
Posterior CC	1.079 ± 0.119	1.054 ± 0.077	0.39

### ADC values and time after birth

**Fig 2:**
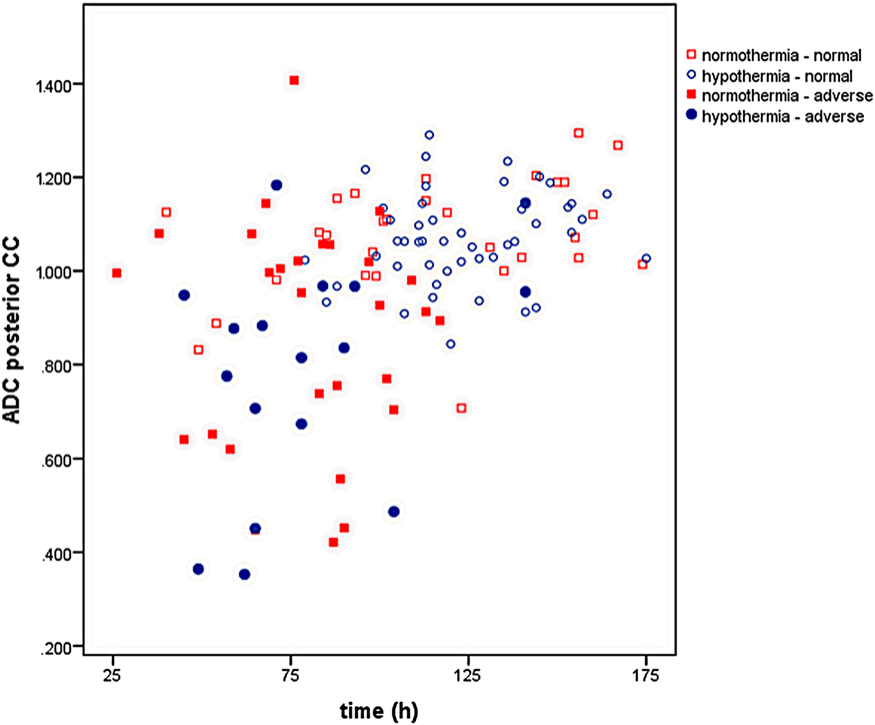
ADC values (x 10^-3^ mm^2^/s) for normothermic and hypothermia-treated infants versus time after birth. Infants with an abnormal outcome are indicated with closed symbols.

ADC values of the posterior part of the CC, temperature regimen, outcome, and time after birth are presented in Fig 2.

### Differences between anterior and posterior ADC values

ADC values of the posterior part of the CC were lower than of the anterior part (mean difference 0.050 x 10^-3^ mm^2^/s, 95% confidence interval of the difference: 0.024 to 0.076 x 10^-3^ mm^2^/s). These differences in the normothermia group were larger than in the hypothermia group (p = 0.01; data not shown).

### ADC values of the CC and MRI patterns

**Fig 3:**
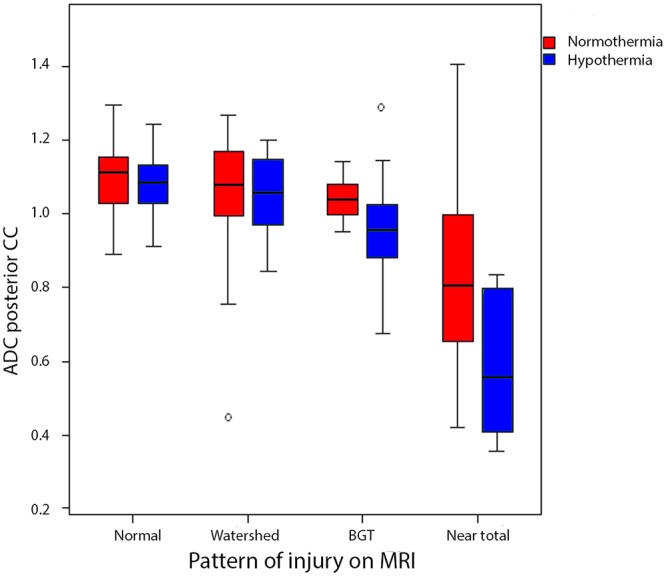
Box-and-whisker plots of ADC values (x 10^-3^ mm^2^/s) of the posterior corpus callosum versus pattern of MRI injury (normal, primary watershed lesions, primary basal ganglia/thalamus lesion, ‘near- total’ brain injury). After modelling, significantly lower ADC values were demonstrated in the infants with ‘near- total’ brain injury in both temperature groups (p<0.001), and in the infants treated with hypothermia and basal ganglia/thalamus injury (p<0.05) compared to infants with a normal MRI. BGT: basal ganglia and thalamus. Dark red box: normothermia; light blue box: hypothermia. Box indicates 25^th^ centile, median, 75^th^ centile, whiskers 5^th^ and 95^th^ centile.

ADC values were higher in infants with a normal MRI or watershed lesions, and were lowest in infants with ‘near-total’ brain injury (Fig 3). Results of the model demonstrated significantly lower ADC values in the infants with ‘near-total’ brain injury in both temperature groups (p<0.001), and in the infants treated with hypothermia and basal ganglia/thalamus injury (p<0.05).

### ADC values of the posterior CC and outcome

**Fig 4:**
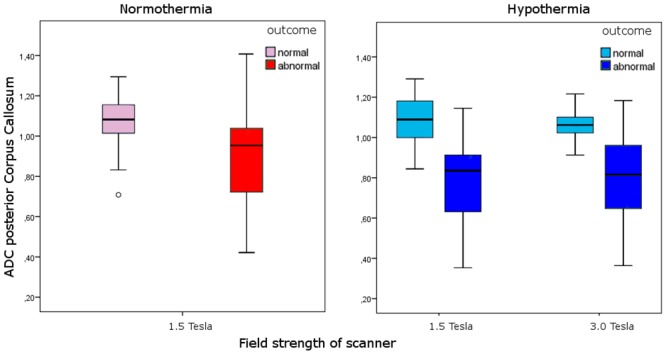
Box-and-whisker plots of ADC values (x 10^-3^ mm^2^/s) of the posterior corpus callosum versus outcome group (normal vs. death or abnormal neurodevelopment), temperature regimen, and field strength of MR system. Infants with an adverse outcome had significantly lower ADC values (p<0.001) after normothermia or hypothermia at both 1.5 and 3.0 T. Light red box: normothermia—normal outcome, dark red box: normothermia—abnormal outcome; light blue box: hypothermia—normal outcome, dark blue box: hypothermia—abnormal outcome.

ADC values of infants treated with hypothermia and normothermia were analyzed separately (Fig 4). In both temperature regimens significantly lower ADC values were found in infants with an adverse outcome. Details are provided in Table 3.

**Table 3:** Field strength, ADC values (x 10^-3^ mm^2^/s) of the posterior part of the corpus callosum, temperature regimen, and outcome.

	Good outcome	Adverse outcome	P-value
Normothermia			
1.5 T	1.075 ± 0.125	0.881 ± 0.242	0.001
Hypothermia			
1.5 T	1.083 ± 0.113	0.774 ± 0.272	0.024
3.0 T	1.054 ± 0.077	0.781 ± 0.245	0.004

### ROC analysis of ADC values of the posterior CC

ROC analysis of infants treated with normothermia showed an Area Under the Curve (AUC) of 0.78 with a cut-off value of 1.024 x 10^-3^ mm^2^/s. In infants with hypothermia data obtained at 1.5 and 3.0T were combined. AUC was 0.87 with a cut-off value of 0.969 x 10^-3^ mm^2^/s.

## Discussion

In the present study we could confirm the hypothesis that ADC values in the CC were lower in term infants with perinatal asphyxia and severe MRI abnormalities compared to those with normal MRI findings. Infants treated with hypothermia had a significantly better outcome which is in agreement with previous findings.[18] ROC analysis demonstrated that a lower cut-off value for prediction of a poor outcome was seen in infants treated with hypothermia compared to those treated with normothermia. This has been demonstrated for other brain areas by others.[11] The signal changes in the CC might be the result of lesions elsewhere in the brain, as reported previously in pre-Wallerian degeneration.[19, 20]

Our findings are in agreement with others who suggested that restricted diffusion within the splenium of the CC of term infants with hypoxic-ischemic encephalopathy appears to be an early neuroradiological marker of adverse outcome.[6, 7]

In a recent study of 20 patients Ancora et al. demonstrated lower ADC values in the CC of the 6 with a poor outcome which supports our findings.[21] Twomey et al. stated that restricted diffusion of either the anterior or posterior CC or both was not found to be predictive of long-term outcome.[22] In their paper they did not measure ADC values in the CC which may have been the reason that in their setting callosal changes were not predictive of an adverse outcome.

In our study all infants with an ADC decrease of the CC had MRI abnormalities elsewhere in the brain, and no infant had abnormalities in the CC only.

Although changes in the CC may be visually apparent, we decided to measure ADC values. Recently we have reported for the basal ganglia and thalamus that ADC measurements can be performed reliably [9], and tools to do so are available on all modern MR scanners. However, normal values of ADC may depend on several variables, including field strength, type of head coil, *b*-values used, and temperature of the patient when scanned during hypothermia.[23, 24] In the present study, in infants examined at 1.5 T a *b*-value of 1000 sec/mm^2^ was used, and in infants examined at 3.0 T a *b*-value of 800 sec/mm^2^. In infants with a normal outcome no significant differences were demonstrated between data obtained at 1.5 T or 3.0 T, thereby enabling us to combine the 1.5 T and 3.0 T results for further analysis.

The usefulness of standard MR imaging has been demonstrated previously in randomized controlled trials.[25, 26] Therapeutic hypothermia modified the association between low ADC values of the CC and an adverse outcome only slightly, confirming the use of MRI as a biomarker after term asphyxia with or without cooling. It is of interest that lower ADC values were needed to result in an adverse outcome supporting the neuroprotective properties of hypothermia. Even though mortality was comparable, fewer infants survived with an adverse outcome following hypothermia. The association between perinatal insults, abnormalities of the CC and an adverse outcome has been demonstrated in older children and experimental animals which corroborates our findings.[4, 27–31]

There are limitations to the present study. First of all, the most seriously affected infants could not be examined because they were too ill to be transferred to the MR system. We do not expect that this has influenced our findings, since we have shown serious abnormalities on cranial ultrasound scans which would most likely have correlated with an abnormal MRI, including changes of the CC.[32] Secondly, infants were examined at different field strengths and with different scanning protocols including different DWI *b*-values. However, this did not appear to have affected our results (see above). Thirdly, most of our infants studied are relatively young, but it is unlikely that they will develop CP when they appear to be normal at 18 months of age. The long-term neurodevelopment and especially the long-term effect of CC involvement with regard to specific CC related function is still unknown and needs to be evaluated. Finally, there was a 14 hour difference in postnatal age of MRI between the normothermia and hypothermia groups due to scanning most of the hypothermia cases after rewarming. It has been demonstrated that ADC values are dependent on time after the hypoxic-ischemic insult, and that the time to pseudonormalization following hypothermia may be delayed.[11] We do not think that this has influenced our present findings, since Rutherford et al.[10] in normothermic infants and Bednarek et al.[11] in normothermic and hypothermic infants have demonstrated that pseudonormalization is a gradual process between 98 and 112 hours after the insult, which is in agreement with our own experience in infants with neonatal stroke.[33] This time difference between normothermia and hypothermia cases was even smaller (8 hours) in the infants with MRI abnormalities in the basal ganglia/thalamus or ‘near- total’ brain injury. Based on the equations in the paper by Bednarek et al. [11] and our own experience the difference in ADC values of an ischemic area between 98 and 112 hours after the insult is 0.040 x 10^-3^ mm^2^/s. With the relatively slow pseudonormalization of ADC values in the most severely affected cases which is even slower during hypothermia, it is unlikely that the difference in ADC values between the infants with a good and a poor outcome has been caused by the postnatal age of scanning.

In addition, infants with an adverse outcome were scanned earlier than those with a good outcome. Our standard policy is to scan between days 4 and 7 after birth, but when redirection of care is considered, MRI examinations are performed as soon as clinically possible. The effects of this difference in age at MRI on our findings are very limited, since pseudonormalization of ADC values of ischemic lesions after hypothermia takes longer than in infants with normothermia. According to the formula provided by Bednarek et al. ADC values of ischemic lesions following hypothermia would have increased from 0.50 to 0.58 x 10^-3^ mm^2^/s between 80 and 120 hours after birth. It is therefore unlikely that this would affect the results of our study.

In conclusion, low ADC values of the CC in full term and near term infants with severe perinatal asphyxia were associated with a poor outcome at 18 months of age. This association persisted in infants with perinatal asphyxia and therapeutic hypothermia, but cut-off ADC values associated with a poor outcome were lower after treatment with hypothermia.

## Supporting Information

S1 Dataset This file contains the main data of the study. (CSV)
